# Incidence and clearance of oral human papillomavirus infection: A population-based cohort study in rural China

**DOI:** 10.18632/oncotarget.16306

**Published:** 2017-03-17

**Authors:** Chaoting Zhang, Fangfang Liu, Yaqi Pan, Qiuju Deng, Xiang Li, Zhonghu He, Mengfei Liu, Tao Ning, Chuanhai Guo, Yongmei Liang, Ruiping Xu, Lixin Zhang, Hong Cai, Yang Ke

**Affiliations:** ^1^ Key Laboratory of Carcinogenesis and Translational Research, Ministry of Education, Laboratory of Genetics, Peking University Cancer Hospital & Institute, Beijing 100142, China; ^2^ Anyang Cancer Hospital, Anyang 455000, China

**Keywords:** oral HPV, natural history, China

## Abstract

The natural history of oral human papillomavirus (HPV) infection which is linked with the increased incidence of oropharyngeal squamous cell cancer (OPSCC) has been incompletely studied. Oral swab specimens and questionnaire data were obtained bi-annually for up to 6 visits from 4314 healthy adults aged 25-69 in rural Anyang, China. HPV infection status was evaluated with PCR-based sequencing. Participants with at least two consecutive valid HPV results within the study period were included in the incidence and clearance analysis. Among 3289 participants included in this analysis (median follow-up time 18.3 months), incidence rates of mucosal HPV, oncogenic mucosal HPV and cutaneous HPV were 0.53 (95% CI: 0.39-0.73), 0.30 (95% CI: 0.20-0.46), and 4.17 (95% CI: 3.70-4.70) per 1,000 person-months respectively. Most newly acquired infections were cleared within one year. Recent practice of oral sex increased the risk of incident infection with mucosal HPV (Adjusted HR, 5.03; 95% CI, 1.16-21.73) and oncogenic mucosal HPV (Adjusted HR, 10.13; 95% CI, 2.14-48.06). Newly acquired oral mucosal HPV infections are rare and most are cleared within one year in rural Chinese. This study expands understanding of the natural history of oral HPV in countries with a lower incidence of HPV-OPSCC.

## INTRODUCTION

Oral human papillomavirus (HPV) infection (primarily HPV-16) is linked with the rapid increase in incidence of oropharyngeal squamous cell cancer (OPSCC) for which there is no demonstrably effective methods of prevention in some developed regions of the world [1]. In China, although the trend of incidence of HPV-related OPSCC has not been systematically analyzed, a recent study showed that HPV infection was detected in 17% of OPSCC in China [2], similar to the detection in 18-36% of OPSCC reported in studies from developed countries [3].

In light of the critical role of oral HPV infection in OPSCC, understanding its natural history is an important public health issue. Previous longitudinal studies, undertaken mainly in developed areas, have shown that oral HPV infection appears to have a low incidence rate and a high clearance rate [4–20]. However, these studies had short follow-up, small sample sizes, and were focused on specific populations (e.g. HIV-infected individuals) or conducted with inclusion of a single gender only [4–20], limiting their ability to adequately estimate incidence and clearance rates of oral HPV infection and identify associated risk factors.

Previously, we reported an overall oral mucosal HPV prevalence of 0.67% in 5410 individuals aged 25-65 years in rural China [21]. This current prospective cohort study was conducted to investigate the incidence and clearance of oral HPV infection, and evaluate potential risk factors for this infection in the same general unvaccinated population.

## RESULTS

A total of 4314 individuals were enrolled in the current study, and the percentage of follow-up at each visit ranged from 57.5% to 70.3% with an average of 66.0%. The human β-globin gene was positive in 98.0% (16759 visits / 17093 visits) of specimens, and 4299 out of 4314 (99.7%) participants had at least one valid HPV result.

As shown in Table 1, 3289 of 4314 participants (76.2%) provided valid specimens at two or more consecutive visits with a median follow-up time of 18.3 months (IQR: 12.2-36.5 months), and were included in the incidence analysis. The median age of the 3289 subjects was 48 years (IQR: 42-59 years). 41.4% of these subjects were men, 90.4% were married, 87.4% had an education level of junior high school or below, and 68.0% were engaged in farming. More than 70.0% of these individuals had never smoked cigarettes or consumed alcohol. A small percentage of individuals had ≥ 2 lifetime sexual partners (3.4%). Almost one third of participants reported preferring hot meals (37.3%). More than half of these individuals reported never brushing teeth (54.6%). Values of time-updated variables among all participants with valid HPV results enrolled at each visit except for the baseline visit were relatively stable over time, as shown in Table 2.

**Table 1:** Fixed baseline characteristics of individuals included and not included in the incidence analysis of oral HPV infection in rural China, 2012-2015

Variables	All participants^a^	Participants included in the incidence analysis^b^	Participants NOT included in the incidence analysis	*P* value^c^
	n (%)	n (%)	n (%)	
	N=4314	N=3289	N=1025	
Follow-up time (months)				
Median (IQR)	12.2 (6.1-24.3)	18.3 (12.2-36.5)	-	-
Age (years)				
Median (IQR)	47 (40-58)	48 (42-59)	42 (35-50)	
25-40	1148 (26.6)	694 (21.1)	454 (44.3)	
41-55	1869 (43.3)	1493 (45.4)	376 (36.7)	
56-69	1297 (30.1)	1102 (33.5)	195 (19.0)	< 0.001
Gender				
Female	2352 (54.5)	1928 (58.6)	424 (41.4)	
Male	1962 (45.5)	1361 (41.4)	601 (58.6)	< 0.001
Education level				
Illiteracy, <1 year	642 (14.9)	557 (16.9)	85 (8.3)	
Primary school, 1-6 years	1160 (26.9)	909 (27.6)	251 (24.5)	
Junior high school, 7-9 years	1932 (44.8)	1408 (42.8)	524 (51.1)	
Senior high school or above, >9 years	358 (8.3)	269 (8.2)	89 (8.7)	
Unknown^d^	222 (5.2)	146 (4.4)	76 (7.4)	< 0.001
Marital status				
Married or cohabiting	3881 (90.0)	2972 (90.4)	909 (88.7)	
Never married, or divorced, separated or widowed	224 (5.2)	178 (5.4)	46 (4.5)	
Unknown^d^	209 (4.8)	139 (4.2)	70 (6.8)	0.371
Types of employment				
Farming in local area	2705 (62.7)	2233 (67.9)	472 (46.1)	
Working in local area	613 (14.2)	513 (15.6)	100 (9.8)	
Working outside local area	430 (10.0)	248 (7.5)	182 (17.8)	
Other	327 (7.6)	177 (5.4)	150 (14.6)	
Unknown^d^	239 (5.5)	118 (3.6)	121 (11.8)	< 0.001
Cigarette smoking^e^				
Never	2985 (69.2)	2362 (71.8)	623 (60.8)	
Former	218 (5.1)	173 (5.3)	45 (4.4)	
Current	1022 (23.7)	716 (21.8)	306 (29.9)	
Unknown^d^	89 (2.1)	38 (1.2)	51 (5.0)	< 0.001
Alcohol consumption^f^				
Never	3001 (69.6)	2459 (74.8)	542 (52.9)	
Former	121 (2.8)	94 (2.9)	27 (2.6)	
Current	778 (18.0)	540 (16.4)	238 (23.2)	
Unknown^d^	414 (9.6)	196 (6.0)	218 (21.3)	< 0.001
Temperature preference for meals				
Cool or moderate	2425 (56.2)	1862 (56.6)	563 (54.9)	
Hot	1531 (35.5)	1228 (37.3)	303 (29.6)	
Unknown^d^	358 (8.3)	199 (6.1)	159 (15.5)	0.012
Frequency of tooth brushing				
0 /week	2253 (52.2)	1797 (54.6)	456 (44.5)	
1-3 /week	809 (18.8)	619 (18.8)	190 (18.5)	
≥4 /week	1028 (23.8)	789 (24.0)	239 (23.3)	
Unknown^d^	224 (5.2)	84 (2.6)	140 (13.7)	0.054
Number of lifetime sex partners				
0-1	3783 (87.7)	3023 (91.9)	760 (74.2)	
≥2	197 (4.6)	111 (3.4)	86 (8.4)	
Unknown^d^	334 (7.7)	155 (4.7)	179 (17.5)	< 0.001

Abbreviation: HPV, human papillomavirus; IQR, interquartile range.

^a^ Participants completed at least one visit.

^b^ Participants having valid HPV results for at least two consecutive visits were included in the incidence analysis.

^c^ Chi-square test was used to compare between participants included in the incidence analysis and not included.

^d^ Unknown was not included in chi-square test.

^e^ Cigarette smoking was defined as at least one cigarette per day for ≥ 12 months.

^f^ Alcohol consumption was defined as consumption of Chinese liquor two or more times per week for ≥ 12 months.

**Table 2:** Time-updated variables^a^ for participants enrolled at each follow-up visit of oral HPV cohort with valid HPV results in rural China, 2012-2015

Variables	Participants enrolled at the second visit, N=2968	Participants enrolled at the third visit, N=2823	Participants enrolled at the fourth visit, N=2899	Participants enrolled at the fifth visit, N=3034	Participants enrolled at the sixth visit, N=2890
Number of recent sex partners					
0	0 (0.0)	794 (28.1)	704 (24.3)	911 (30.0)	1015 (35.1)
≥1	0 (0.0)	2011 (71.2)	2175 (75.0)	2115 (69.7)	1868 (64.6)
Unknown^b^	2968 (100.0)	18 (0.6)	20 (0.7)	8 (0.3)	7 (0.2)
Frequency of recent sexual intercourse					
≤2 /month	0 (0.0)	1897 (67.2)	2044 (70.5)	2172 (71.6)	2155 (74.6)
>2 /month	0 (0.0)	876 (31.0)	834 (28.8)	802 (26.4)	650 ( 22.5)
Unknown^b^	2968 (100.0)	50 (1.8)	21 (0.7)	60 (2.0)	85 (2.9)
Recent practice of oral sex					
No	2921 (98.4)	2784 (98.6)	2837 (97.9)	2968 (97.8)	2853 (98.7)
Yes	27 (0.9)	31 (1.1)	51 (1.8)	57 (1.9)	30 (1.0)
Unknown^b^	20 (0.7)	8 (0.3)	11 (0.4)	9 (0.3)	7 (0.2)
Recent open-mouth kissing					
No	0 (0.0)	2613 (92.6)	2612 (90.1)	2763 (91.1)	2668 (92.3)
Yes	0 (0.0)	202 (7.2)	276 (9.5)	262 (8.6)	215 (7.4)
Unknown^b^	2968 (100.0)	8 (0.3)	11 (0.4)	9 (0.3)	7 (0.2)
History of recent gingival bleeding					
No	2200 (74.1)	2075 (73.5)	2199 (75.9)	2446 (80.6)	2276 (78.8)
Yes	748 (25.2)	740 (26.2)	689 (23.8)	580 (19.1)	607 (21.0)
Unknown^b^	20 (0.7)	8 (0.3)	11 (0.4)	8 (0.3)	7 (0.2)
History of recent oral ulcer					
No	2502 (84.3)	2424 (85.9)	2545 (87.8)	2725 (89.8)	2530 (87.5)
Yes	446 (15.0)	391 (13.9)	343 (11.8)	301 (9.9)	353 (12.2)
Unknown^b^	20 (0.7)	8 (0.3)	11 (0.4)	8 (0.3)	7 (0.2)
Number of recent decayed teeth					
0	2502 (84.3)	1924 (68.2)	2097 (72.3)	2166 (71.4)	2364 (81.8)
≥1	446 (15.0)	891 (31.6)	791 (27.3)	860 (28.4)	519 (18.0)
Unknown^b^	20 (0.7)	8 (0.3)	11 (0.4)	8 (0.3)	7 (0.2)
Number of recent missing teeth					
0	0 (0.0)	2639 (93.5)	2775 (95.7)	2888 (95.2)	2652 (91.8)
≥1	0 (0.0)	176 (6.2)	113 (3.9)	134 (4.4)	137 (4.7)
Unknown^b^	2968 (100.0)	8 (0.3)	11 (0.4)	12 (0.4)	101 (3.5)
Recent self-rated oral health					
Healthy or Fair	0 (0.0)	0 (0.0)	2790 (96.2)	2809 (92.6)	2480 (85.8)
Unhealthy	0 (0.0)	0 (0.0)	98 (3.4)	217 (7.2)	403 (13.9)
Unknown^b^	2968 (100.0)	2823 (100.0)	11 (0.4)	8 (0.3)	7 (0.2)
Recent self-rated health					
Healthy or Fair	0 (0.0)	2568 (91.0)	2711 (93.5)	2745 (90.5)	2555 (88.4)
Unhealthy	0 (0.0)	247 (8.8)	177 (6.1)	281 (9.3)	327 (11.3)
Unknown^b^	2968 (100.0)	8 (0.3)	11 ( 0.4)	8 (0.3)	8 (0.3)

Abbreviation: HPV, human papillomavirus.

^a^ Time-updated variables refer to the six-month period prior to the follow-up.

^b^ Unknown, denoted that the indicated data was not collected.

The overall incidence rates of mucosal HPV, oncogenic mucosal HPV, and nononcogenic mucosal HPV were 0.53 (95% CI: 0.39-0.73), 0.30 (95% CI: 0.20-0.46), and 0.23 (95% CI: 0.14-0.37) per 1,000 person-months (PMs) respectively (Table 3). During the first 12 months of follow-up, 0.64% (95% CI: 0.37-0.92) of individuals acquired an incident mucosal HPV infection, 0.34% (95% CI: 0.14-0.53) acquired an oncogenic mucosal HPV infection, and 0.31% (95% CI: 0.12-0.49) acquired a nononcogenic mucosal HPV. HPV-45 had the highest incidence rate among the 7 types of oncogenic mucosal HPV which were detected, followed by HPV-58 and HPV-16. The most commonly detected incident nononcogenic mucosal HPV infection was HPV-90, followed by HPV-11 and HPV-6 (Table 3). The overall incidence of cutaneous HPV was 4.17 (95% CI: 3.70-4.70) per 1000 PMs. Oral HPV-3 was the most frequently acquired cutaneous HPV type, followed by types 57 and 94.

**Table 3:** Incidence and clearance of oral HPV infection among participants from rural China, 2012-2015

HPV type	Incident cases^a^ N=3289	Person-months	Incidence rate, 95% CI^b^ (per 1000 person-months)	12-month incidence (95% CI)	Newly acquired infections^c^	Cleared infections^d^	Median time to clearance, 95% CI(months)
Any	297	63578	4.67 (4.17-5.23)	5.19% (4.42-5.96)	225	219	6.08 (6.08-6.08)
Mucosal	37	69571	0.53 (0.39-0.73)	0.64% (0.37-0.92)	29	29	6.08 (6.08-6.08)
Oncogenic	21	70043	0.30 (0.20-0.46)	0.34% (0.14-0.53)	16	16	6.08 (6.08-6.08)
HPV-16	4	70380	0.06 (0.02-0.15)	0.06% (0.00-0.15)	3	3	6.08 (6.08-NE)
HPV-18	2	70441	0.03 (0.01-0.11)	0.06% (0.00-0.15)	1	1	NE
HPV-26	1	70441	0.01 (0.002-0.10)	0.03% (0.00-0.09)	1	1	NE
HPV-45	6	70374	0.09 (0.04-0.19)	0.12% (0.00-0.24)	6	6	6.08 (6.08-NE)
HPV-52	2	70414	0.03 (0.007-0.11)	0.03% (0.00-0.09)	2	2	6.08 (NE-NE)
HPV-58	5	70356	0.07 (0.03-0.17)	0.03% (0.00-0.09)	2	2	6.08 (NE-NE)
HPV-68	1	70411	0.01 (0.002-0.10)	0.00%	1	1	NE
Non-oncogenic	16	69991	0.23 ( 0.14-0.37)	0.31% (0.12-0.49)	13	13	6.08 (NE-NE)
HPV-6	3	70423	0.04 (0.01-0.13)	0.09% (0.00-0.19)	2	2	6.08 (NE-NE)
HPV-11	5	70271	0.07 (0.03-0.17)	0.09% (0.00-0.19)	5	5	6.08 (NE-NE)
HPV-43	2	70447	0.03 (0.01-0.11)	0.03% (0.00-0.09)	1	1	NE
HPV-62	1	70453	0.01 (0.002-0.10)	0.00%	1	1	NE
HPV-90	5	70265	0.07 (0.03-0.17)	0.09% (0.00-0.19)	4	4	6.08 (NE-NE)
Cutaneous	268	64332	4.17 (3.70-4.70)	4.66% (3.93-5.39)	196	190	6.08 (6.08-6.08)
HPV-3	126	67280	1.87 (1.57-2.23)	2.08% (1.58-2.57)	89	87	6.08 (6.08-6.08)
HPV-7	1	70441	0.01 (0.002-0.10)	0.00%	1	1	NE
HPV-10	18	69955	0.26 (0.16-0.41)	0.40% (0.18-0.61)	13	13	6.08 (6.08-9.12)
HPV-27	3	70423	0.04 (0.01-0.13)	0.06% (0.00-0.15)	2	2	6.08 (NE-NE)
HPV-28	1	70460	0.01 (0.002-0.10)	0.00%	0	0	NE
HPV-29	7	70311	0.10 (0.05-0.21)	0.15% (0.02-0.29)	4	4	6.08 (6.08-NE)
HPV-57	72	69109	1.04 (0.83-1.31)	1.10% (0.74-1.46)	50	48	6.08 (6.08-6.08)
HPV-75	19	70003	0.27 (0.17-0.43)	0.34% (0.14-0.53)	15	15	6.08 (6.08-NE)
HPV-76	1	70432	0.01 (0.002-0.10)	0.03% (0.00-0.09)	1	1	NE
HPV-94	28	69903	0.40 (0.28-0.58)	0.58% (0.32-0.84)	21	19	6.08 (6.08-9.12)
Vaccine (6, 11, 16, 18)	14	70128	0.20 (0.12-0.34)	0.30% (0.12-0.49)	11	11	6.08 (6.08-6.08)

Abbreviation: HPV, human papillomavirus; CI, confidence interval; NE, not estimable.

^a^ Participants with at least two consecutive valid HPV results within the study period and a negative test for a specific type of HPV at the time of enrollment in this study were included in the incidence analysis. For incidence rate calculation person-time was defined as the time from study entry to the first HPV positive visit.

^b^ The calculation of 95% CIs for incidence rate was based on the number of events modeled as a Poisson variable for total person-months.

^c^ Multiple incident infections in one participant were treated separately in the clearance analysis. Incident infections detected at a participant's last visit were not included for clearance analysis. The median time estimates as well as 95% CIs for such grouped HPV types were calculated using the clustered Kaplan-Meier method.

^d^ An HPV clearance event was defined as a single negative result for a specific HPV type after testing positive.

In the clearance analysis, 26 out of 29 (89.7%) newly acquired mucosal HPV infections were cleared within six months and another 3 mucosal HPV infections were cleared within one year. For cutaneous HPV, 143 out of 196 (73.0%) newly acquired infections were cleared within six months, and 185 out of 196 (94.4%) were cleared within one year. With regard to type specificity, the estimated median clearance time for all HPV types was about six months (Table 3).

No significant difference in the incidence of oral mucosal HPV infection was observed across age groups and gender (Table 4). Similar age and gender patterns were also observed for the acquisition of oncogenic mucosal HPV infection. Number of lifetime sex partners was marginally associated with acquisition of oncogenic mucosal HPV infection in univariate analysis (Crude HR, 4.13; 95% CI, 0.95-17.95) (Table 4). Number of recent sex partners had a statistically significant association with acquisition of oncogenic mucosal HPV infection in univariate analysis (Crude HR, 7.66; 95% CI, 1.02-57.65) and this association was marginal in multivariate analysis (Adjusted HR, 7.08; 95% CI, 0.86-58.20). Recent practice of oral sex conferred a greater risk for acquisition of mucosal HPV (Adjusted HR, 5.03; 95% CI, 1.16-21.73) and oncogenic mucosal HPV (Adjusted HR, 10.13; 95% CI, 2.14-48.06) in both univariate and multivariate analysis (Table 4).

**Table 4:** Univariate and multivariate analyses of factors associated with incidence of oral HPV infection among individuals from rural China, 2012-2015

Variables	Incidence of HPV infection			
	Mucosal HPV		Oncogenic mucosal HPV	
	Crude HR^a^	Adjusted HR^b^	Crude HR^a^	Adjusted HR^c^
	(95% CI)	(95% CI)	(95% CI)	(95% CI)
Age^d^ (years)				
25-40	1.00	1.00	1.00	1.00
41-55	1.56 (0.58-4.17)	1.70 (0.63-4.57)	2.49 (0.56-11.06)	2.91 (0.64-13.29)
56-75	1.16 (0.41-3.31)	1.33 (0.46-3.86)	1.41 (0.28-7.02)	2.33 (0.39-13.97)
*P*_trend_^e^	0.998		0.968	
Gender^d^				
Female	1.00	1.00	1.00	1.00
Male	0.88 (0.45-1.74)	0.93 (0.47-1.84)	1.24 (0.52-2.94)	1.53 (0.58-4.02)
Marital status^d^				
Married or cohabiting	1.00		1.00	
Never married, divorced, separated or widowed	0.47 (0.06-3.46)		NA	
Education level^d^				
Illiteracy, <1 year	1.00		1.00	
Primary school, 1-6 years	1.60 (0.50-5.10)		1.71 (0.45-6.45)	
Junior high school, 7-9 years	2.15 (0.73-6.33)		1.22 (0.32-4.60)	
Senior high school or above, >9 years	1.20 (0.22-6.53)		0.81 (0.08-7.80)	
*P*_trend_^e^	0.321		0.842	
Type of employment^d^				
Farming in local area	1.00		1.00	
Working in local area	0.63 (0.22-1.80)		0.52 (0.12-2.27)	
Working outside local area	NA		NA	
Other	2.64 (0.92-7.59)		2.29 (0.52-10.05)	
*P*_trend_^e^	0.682		0.989	
Cigarette smoking^d, f^				
Never	1.00		1.00	
Former	1.62 (0.49-5.35)		2.96 (0.86-10.22)	
Current	0.86 (0.35-2.09)		0.78 (0.23-2.70)	
*P*_trend_^e^	0.856		0.954	
Alcohol consumption^d, g^				
Never	1.00		1.00	
Former	1.81 (0.43-7.58)		3.42 (0.79-14.86)	
Current	0.52 (0.16-1.71)		0.65 (0.15-2.84)	
*P*_trend_^e^	0.363		0.801	
Temperature preference for meals^d^				
Cool or moderate	1.00		1.00	
Hot	1.09 (0.56-2.13)		1.17 (0.49-2.83)	
Frequency of tooth brushing^d^				
0 /week	1.00		1.00	
1-3 /week	0.99 (0.39-2.51)		1.11 (0.35-3.55)	
≥4 /week	1.69 (0.82-3.47)		1.53 (0.58-4.01)	
*P*_trend_^e^	0.178		0.402	
Number of lifetime sex partners^d^				
0-1	1.00		1.00	
≥2	2.16 (0.52-9.03)		4.13 (0.95-17.95)	
Number of recent sex partners^h^				
0	1.00		1.00	1.00
≥1	1.98 (0.82-4.81)		7.66 (1.02-57.65)	7.08 (0.86-58.20)
Frequency of recent sexual intercourse^h^				
≤2 /month	1.00		1.00	
>2 /month	1.36 (0.64-2.89)		2.24 (0.85-5.92)	
Recent practice of oral sex^h^				
No	1.00	1.00	1.00	1.00
Yes	4.74 (1.14-19.74)	5.03 (1.16-21.73)	8.72 (2.03-37.55)	10.13 (2.14-48.06)
Recent open-mouth kissing^h^				
No	1.00		1.00	
Yes	1.97 (0.76-5.10)		2.22 (0.64-7.69)	
History of recent gingival bleeding^h^				
No	1.00		1.00	
Yes	1.52 (0.77-3.04)		1.27 (0.49-3.28)	
History of recent oral ulcer^h^				
No	1.00		1.00	
Yes	0.18 (0.03-1.33)		0.33 (0.04-2.48)	
Number of recent decayed teeth^h^				
0	1.00		1.00	
≥1	1.18 (0.57-2.44)		0.98 (0.36-2.69)	
Number of recent missing teeth^h^				
0	1.00		1.00	
≥1	0.60 (0.08-4.43)		1.11 (0.15-8.34)	
Recent self-rated oral health^h^				
Healthy or Fair	1.00		1.00	
Unhealthy	0.50 (0.07-3.76)		0.84 (0.11-6.52)	
Recent self-rated health^h^				
Healthy or Fair	1.00		1.00	
Unhealthy	0.31 (0.04-2.27)		0.57 (0.08-4.32)	

Abbreviation: HPV, human papillomavirus; HR, hazard ratio; CI, confidence interval; NA, not available.

^a^ Univariate analysis was conducted using an extended Cox model with robust variance.

^b^ Multivariate analysis was conducted using an extended Cox model with robust variance adjusted for age, gender and recent practice of oral sex.

^c^ Multivariate analysis was conducted using an extended Cox model with robust variance adjusted for age, gender, number of recent sex partners and recent practice of oral sex.

^d^ Time invariant, measured at baseline.

^e^ *P* values for trend were calculated by treating categorical variables as continuous variables.

^f^ Cigarette smoking was defined as at least one cigarette per day for ≥ 12 months.

^g^ Alcohol consumption was defined as consumption of Chinese liquor two or more times per week for ≥ 12 months.

^h^ Time-updated, refers to the 6-month period prior to the follow-up.

## DISCUSSION

To our knowledge, this is the largest population-based study investigating incidence and clearance of oral HPV infection in both men and women. Data from this study demonstrates that acquisition of oral mucosal HPV infection (and especially oncogenic mucosal HPV) is a rare event, and most incident infections are cleared within one year in rural China. The incidence rate of mucosal HPV infection is similar across gender and age groups. Recent performance of oral sex was predominated among characteristics of sexual behaviors affecting acquisition of mucosal HPV infection. This study expands our understanding of natural history of oral HPV in countries with a lower incidence of HPV-OPSCC.

Previous studies regarding oral HPV incidence were carried out primarily in the USA and European countries [4, 6, 10, 12–20]. For mucosal HPV types, incidence estimates in these studies ranged from 5.6 to 61 per 1,000 PMs [4, 17, 20]. We observed a much lower incidence for mucosal and oncogenic mucosal HPV infection (0.53 and 0.30 per 1000 PMs) in healthy Chinese individuals than in the American general population (5.6 and 2.5 per 1000 PMs) [20]. These results were consistent with the lower prevalence of oral mucosal HPV and oncogenic mucosal HPV in our previous cross-sectional study [21] and the lower incidence of HPV-OPSCC in China [2]. Based on this same general population, two cohort studies investigating the natural history of male genital [22] and female cervical (unpublished data) HPV infection using identical specimen sampling and HPV detection methods were also conducted by our group, which provided us an opportunity to comprehensively interpret the natural history data of HPV infection involving different anatomical sites. Similar to the oral cavity, the incidence of mucosal and oncogenic mucosal HPV infection of penis (8.15 and 4.90 per 1,000 PMs) [22] and cervix (7.15 and 6.27 per 1,000 PMs) was also lower in this same Chinese population than in corresponding western studies [20, 23, 24]. In addition, by comparison of different body sites, we observed that acquisition of oral mucosal HPV infection was much rarer than those in male and female genital sites, supporting the findings reported elsewhere [7, 20]. Our studies provide prospective evidence that both population characteristics and body sites affect the natural history of HPV infection. In terms of population characteristics, sexual behaviors are considerably more conservative and oral sex is relatively uncommon among rural Chinese population as compared with westerners, and this may play a role in the lower incidence of oral HPV infection in this rural Chinese area.

To date, five studies have investigated the prevalence of oral cutaneous HPV infection [11, 21, 25–27], and ours is the first prospective study to investigate the incidence of oral cutaneous HPV infection. We found that the incidence of cutaneous HPV was much higher than mucosal HPV, in keeping with the corresponding data on oral HPV prevalence in cross-sectional studies previously reported by our group [21] and other groups [26]. Although cutaneous HPV types were detected mainly in benign lesions and deemed nononcogenic [28], the interplay between cutaneous HPV and mucosal HPV as well as its pathologic changes remains unknown and warrants further investigation.

In this study, the risk of acquiring mucosal HPV infection was constant across age groups. This is different from age patterns in the prevalence data, which showed peak values at younger and/or older ages [21, 29]. This discrepancy was observed not only in our study population but also in western studies [4, 13, 20], and may reflect the fact that the increased prevalence in specific age groups could be due to increased duration of oral infection rather than increased incidence. With regard to gender, no difference was observed in the incidence of oral mucosal HPV infection among males and females in this study, consistent with our previously reported prevalence data and prospective results seen among healthy U.S. adults [4, 16].

A significantly higher incidence of oral HPV infection was observed in individuals with a recent performance of oral sex, consistent with previous reports indicating that oral sex may be a risk factor for both oral HPV prevalence [30, 31] and HPV-positive head and neck cancer [32]. Among prospective studies having previously evaluated the association of oral sexual behavior and acquisition of oral HPV, similar positive findings were observed by some groups [4, 13, 15]. However, null risk estimates were found by others [10, 17]. These null results may be due to insufficient power, recall bias or inadequate detail in the questionnaire. The data on sexual behavioral characteristics including recent practice of oral sex obtained bi-annually at each visit in this study, may be more accurate (less likely to result in exposure misclassification) than the information collected once only at baseline as in most previous studies. The use of time-updated interview data may increase our ability to precisely determine the association between risky sexual behavior factors and the acquisition of oral HPV infection. In addition to recent practice of oral sex, increase in numbers of recent sex partners was also significantly related to elevated risk of acquiring an oncogenic mucosal HPV infection in univariate analysis, although this association was not statistically significant in multivariate analysis. These findings suggest there may be sexual transmission of HPV infection between oral and genital sites, supporting the observation that oral HPV infection was higher among individuals who either themselves had a prevalent genital infection or had a partner with a genital HPV infection as reported in cross-sectional studies [33, 34]. Nevertheless, more adequately powered prospective studies evaluating HPV infection at both oral and genital sites are needed to clarify the transmission dynamics of oral HPV infection and its associated factors.

Our study showed that the median clearance time of oral HPV infection was around six months and most infections were cleared within one year. Clearance of oral HPV infection was thus similar to that of anogenital HPV infections in healthy populations reported by us and others [7, 20, 22]. However, it must be kept in mind that our definition of clearance which was based on only one negative HPV result may underestimate the true duration of infection. Employing a more stringent definition, such as two consecutive negative HPV results would have increased right-censored data and reduced precision by restricting the number of analyzable visits. Larger studies with more follow-up visits are needed to obtain precise estimates of clearance rates and its associated risk factors.

The limitations of this study are as follows. First, individuals without at least two consecutive visits were not included in incidence and clearance analysis, which may reduce the generalizability of our findings to a wider population. Second, the cell collection method in this study may decrease the detection rate of oral HPV infection. The 2 most common collection methods have been reported, including mouth rinses/gargles [20, 35] and cytobrushes/swabs [21, 36]. Although oral rinse/gargle specimen collection may yield larger amounts and higher molecular weight DNA than cytobrush/swab specimen collection [37], in terms of oral HPV detection, several studies showed low HPV type-specific agreement between these two collection methods and each missed infections detected by the other. In order to maximize the detection of oral HPV types and reflect HPV infection status for the whole oral cavity, using multiple oral exfoliated cell sampling methods such as swabbing together with rinsing may be better [15, 20]. Third, the median time to clearance for oral HPV infection must be interpreted with caution. A six-month sampling interval may result in underestimation of incidence and clearance rates, due to the recognized transience of HPV infection. Fourth, although over 3000 participants were enrolled in this study, the small number of incident infections and cleared infections affected the precision of our assessment of rates and predictors of incidence and clearance. However, this study nonetheless provides essential basic information concerning the natural history of oral HPV in individuals from rural China.

In summary, the incidence of oral mucosal HPV infections is low in rural China, and its risk is increased by recent practice of oral sex. Oral HPV infections typically appear to be transient and most are cleared within one year. These findings not only replenish data on oral HPV natural history in countries with lower incidence of HPV-OPSCC, but may also help inform future efforts in prevention of HPV infection, such as use of HPV vaccination for HPV-OPSCC. Additional research with larger sample size and longer duration of follow-up will be necessary to better understand persistent oral HPV infection as well as factors associated with oral HPV persistence and clearance.

## MATERIALS AND METHODS

### Study population

This population-based study was based on an ongoing esophageal cancer cohort study in rural Anyang, China [38]. The current investigation utilized a sub-cohort including 5 of the 9 target villages which comprised the parent cohort study in 2012-2015. Eligibility criteria for subjects enrolled in this study were as follows: 1) permanent residency in the target villages; 2) age 25-69; 3) no prior diagnosis of tumor, mental disorder, cardiac or cerebrovascular disease; 4) no past history of HBV, HCV, or HIV infection. All cohort members were followed from 2012 to 2015 for up to 6 evaluation visits which took place bi-annually, except for the second visit which was scheduled one year after the first visit. This study was approved by the Institutional Review Board of the Peking University School of Oncology, China. All participants in this study provided written informed consent.

### Specimen and data collection

Exfoliated cells from the oral cavity were collected using saline-moistened cotton swabs as reported previously [21]. Oral specimens were collected from the palate, the gingival surfaces, the buccal mucosa, the inner upper and lower lips, and the top and bottom of the tongue by performing 5 strokes at each site. Specimens were transferred by rotation of the swab into a collection tube containing 1 ml of normal saline. Specimens were centrifuged at 3,000 × g for 10 minutes at 4°C, and the supernatant was decanted and the cell pellet was stored at -70°C pending HPV testing.

A one-on-one computer-aided interview was administered by a trained interviewer. Demographic characteristics, cigarette smoking, alcohol consumption, temperature preference for meals, frequency of tooth brushing and number of lifetime sexual partners were evaluated only at the baseline visit (fixed variables). Number of recent sex partners, frequency of recent sexual intercourse, recent practice of oral sex, recent open-mouth kissing, history of recent gingival bleeding, history of recent oral ulcers, number of recent decayed teeth, number of recent missing teeth, recent self-rated oral health, and recent self-rated health were investigated at each visit except for the baseline visit (time-updated variables refer to the six-month period prior to the follow-up interview).

### Laboratory procedure

Specimen DNA was extracted on a Biomek 3000 automated workstation using the E.Z.N.A.TM Mag-Bind Tissue DNA Kit (Omega Bio-Tek, Inc.). The β-globin gene was evaluated in all specimens by PCR. HPV DNA was detected in valid (β-globin positive) specimens using a highly sensitive PCR primer set (SPF1/GP6+) amplifying a 184-bp fragment of the L1 open-reading frame [21, 39]. HPV types were subsequently identified by direct sequencing of PCR products or by cloning and sequencing. Rigorous quality control procedures were implemented through testing to avoid potential contamination [21].

### Statistical analysis

The incidence analysis was conducted in a type-specific manner and the individual subject was treated as the calculating unit. Participants with at least two consecutive valid HPV results within the study period and a negative test for a specific type of HPV at enrollment were included in the incidence analysis. Incident events in this study were defined as the first HPV positive results for a specific HPV type after enrollment and events were assumed to arise at the mid-point of the interval before the HPV detection. For HPV group (any, mucosal, oncogenic mucosal, nononcogenic mucosal and cutaneous type), only the first incident event of each corresponding HPV group was included in incidence analysis for each subject. Person-time quantification for incidence rate calculation was counted from study entry to the occurrence of an HPV incident event and participants without incident events were censored at their last consecutive visits within the study period. The calculation of 95% CIs for incidence rate was based on the number of events modeled as a Poisson variable for the total person-months [40].

For clearance analysis, HPV infection was regarded as the calculating unit. Only incident infections were included in the clearance analysis and incident events identified at a participant's last visit were excluded. An HPV clearance event was defined as a single negative result for a specific HPV type after the first positive test result and the clearance event was assumed to occur at the mid-point between HPV positive and negative results. Person-time for clearance was calculated from the occurrence of an HPV incident event to HPV clearance or censoring. In estimating median HPV clearance time, one individual might contribute to more than one observation for a given HPV risk group (e.g. mucosal HPV), and the time estimates as well as the 95 % CIs for such groups were therefore calculated using the clustered Kaplan-Meier method adjusting for within subject correlations [41].

Since some factors were time-updated at each visit, factors associated with HPV incidence were assessed using the extended Cox model with robust variance [42]. All fixed and time-updated factors were assessed with univariate analysis. Variables with statistical significance in univariate models and design variables (age and gender) were included in final multivariable models. The proportional hazard assumptions for Cox models were tested and no gross violation was found.

Statistical analysis was conducted using STATA version 12.0 (STATA Corporation, College Station, TX, USA) and R version 3.1.0. All statistical tests were two-sided at the 0.05 significance level.

## Abbreviations

HPV: human papillomavirus
CI: confident interval
HR: hazard ratio
OPSCC: oropharyngeal squamous cell cancer
IQR: interquartile range
PMs: person-months.
